# Electrocatalytic C—N Coupling: Advances in Urea Synthesis and Opportunities for Alternative Products

**DOI:** 10.1002/cssc.202402566

**Published:** 2025-04-12

**Authors:** Parker Ballard‐Kyle, Isabel Hsieh, Huiyuan Zhu

**Affiliations:** ^1^ Department of Chemistry University of Virginia 409 McCormick Rd Charlottesville VA 22904 USA

**Keywords:** catalyst design, C—N coupling, electrosynthesis, urea, urea quantification

## Abstract

Urea is an essential fertilizer produced through the industrial synthesis of ammonia (NH_3_) via the Haber–Bosch process, which contributes ≈1.2% of global annual CO_2_ emissions. Electrocatalytic urea synthesis under ambient conditions via C—N coupling from CO_2_ and nitrogen species such as nitrate (NO_3_^−^), nitrite (NO_2_^−^), nitric oxide (NO), and nitrogen gas (N_2_) has gained interest as a more sustainable route. However, challenges remain due to the unclear reaction pathways for urea formation, competing reactions, and the complexity of the resulting product matrix. This review highlights recent advances in catalyst design, urea quantification, and intermediate identification in the C—N coupling reaction for electrocatalytic urea synthesis. Furthermore, this review explores future prospects for industrial C—N coupling, considering potential nitrogen and carbon sources and examining alternative C—N coupling products, such as amides and amines.

## Graphical abstract

This review explores electrocatalytic urea synthesis via carbon–nitrogen (C—N) coupling from CO_2_ and nitrogen species, specifically nitrate, nitrite, nitric oxide, and nitrogen gas. It discusses recent discoveries in catalyst design, reaction pathways, and detection methods. Future outlooks on industrial applications, alternative C—N coupling products, and challenges in optimizing efficiency, selectivity, and scalability are also provided.

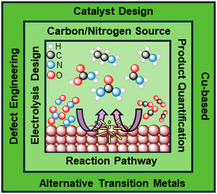

## 1 Introduction

**Figure 1:**
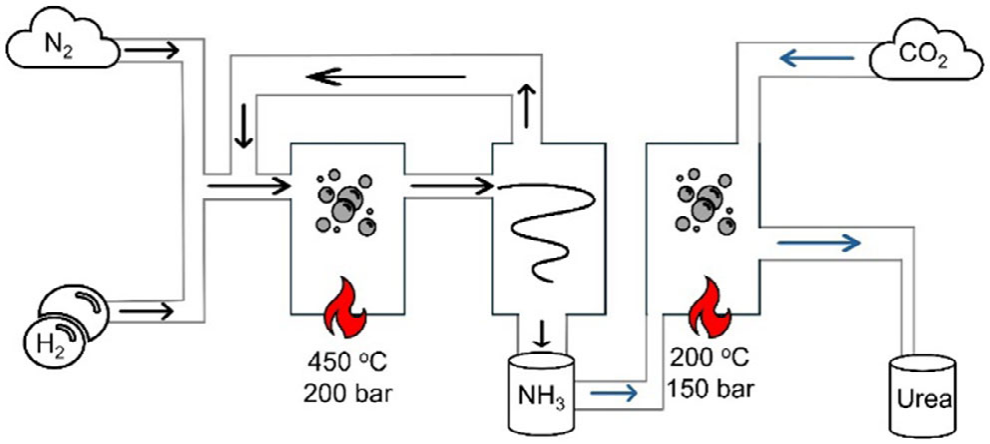
Schematic illustration of the industrial Haber–Bosch process (black arrows) and Bosch–Meiser process (blue arrows) for NH_3_ and urea synthesis.

Nitrogen, alongside carbon, hydrogen, and oxygen, is a key limiting nutrient essential for photosynthesis, phytohormone production, proteomic changes, and overall plant growth and development.^[^ ^1^ ^]^ With the growth of the global population, the demand for an affordable, nitrogen‐rich synthetic fertilizer, produced on an industrial scale, has intensified.^[^ ^2^ ^]^ Anhydrous ammonia (NH_3_), synthesized via Haber–Bosch process (HB), meets these demands (**Figure** **1**).^[^ ^3^ ^]^ Considered one of the most important industrial chemical processes, the HB synthesis combines N_2_ and hydrogen gas (H_2_) (R1), derived from steam reformation, over an iron‐based catalyst at temperatures of 400–500 °C and pressures between 100 and 200 bar.^[^ ^4^ ^]^

(R1) $$N_{2}+3H_{2}\to 2NH_{3}$$

This energy‐intensive reaction accounts for over 1.4% of global CO_2_ emissions and nearly 2% of the world's energy consumption.^[^ ^5^ ^]^

Approximately 80% of the world's NH_3_ production is used for the industrial synthesis of urea (CO(NH_2_)_2_), an organic compound containing 46% nitrogen, with 90% of this essential commodity chemical dedicated to agriculture.^[^ ^6^ ^]^ In the Bosch–Meiser process, the NH_3_ from the HB reacts with CO_2_ at 160–200 °C and 150–250 bar, forming ammonium carbamate (R2), which then decomposes into urea and water (R3).^[^ ^7^ ^]^

(R2) $$\begin{array}{c} 2NH_{3}+{\text{CO}}_{2}\to {\text{NH}}_{2}\text{COON}H_{4} \end{array}$$

(R3) $${\text{NH}}_{2}\text{COON}H_{4}\to \text{CO}{\left(NH_{2}\right)}_{2}+H_{2}O$$

In 2021, global urea production reached 233 million metric tons and is projected to rise to 300 million metric tons by 2030.^[^ ^8^ ^]^ With the world population expected to reach 9.7 billion by 2050, the demand for urea fertilizers will continue to grow, further intensifying pressure on this already energy‐intensive process.^[^ ^2^ ^]^ Consequently, alternative methods, such as electrochemical techniques, for urea production and other commodity chemicals, have attracted interest from researchers.^[^ ^9^ ^]^ Electrochemical processes can be powered by renewable energy sources and operate under ambient conditions, helping to reduce the anthropogenic carbon footprint while bypassing the harsh reaction conditions typically associated with traditional synthetic methods.^[^ ^10^ ^]^ The direct utilization of waste products and pollutants, such as CO_2_ and/or nitrate (NO_3_^−^), allows them to be recycled into the economy as valuable commodities, while simultaneously mitigating greenhouse gas emissions. Research and development of these methods present a more sustainable approach to industrial urea synthesis compared to the HB process. The electrocatalytic carbon‐nitrogen (C—N) coupling reaction between CO_2_ and nitrogen‐containing species, such as NO_3_^−^, nitrite (NO_2_^−^), nitric oxide (NO), and N_2_, offers a promising alternative to the energy‐intensive urea synthesis process. This approach enables the use of an electrolyzer powered by renewable energy sources under ambient conditions, eliminating the need for H_2_ produced from steam reforming, while also recycling waste products back into the economy.^[^ ^11^ ^]^

However, several challenges must be addressed in the electrochemical C—N coupling reaction for urea synthesis in order to scale the process to industrial standards. First, the limited mechanistic understanding of electrocatalytic C—N coupling impedes the efficient design of catalysts, making it difficult to identify the optimal variables for an unknown reaction.^[^ ^11^ ^]^ Second, the strong bond dissociation energy (BDE) of CO_2_ (803 kJ mol^−1^) and nitrogen species complicates the balance between their adsorption and subsequent reactions.^[^ ^12^, ^13^ ^]^ Third, the C—N coupling reaction competes with various other reactions, including the CO_2_ reduction reaction (CO_2_RR), hydrogen evolution reaction (HER), and nitrate reduction reaction (NO_3_RR), which can generate alternative products such as formate, ethanol, ammonium, NO_2_^−^, and other C—N products like amino acids, methylamine, and acetamide. This broad range of potential products further complicates product separation, making the process undesirable for industrial applications.^[^ ^14^, ^15^, ^16^, ^17^ ^]^ This review explores the recent advancements, and sustainability benefits that the C—N coupling reaction may offer. Specifically, it discusses progress toward urea synthesis from various nitrogen sources, along with the corresponding reaction mechanisms of C—N coupling. The advantages and challenges of different nitrogen sources and urea product detection methods are also examined. Additionally, a synthesis of the current catalytic approaches and a review of pathways toward both urea and other C—N products from the discussed reactants are also provided.

## 2 Recent Advances in Catalysts Leveraging Diverse Nitrogen Sources

### 2.1 NO_3_^−^ as the Nitrogen Feedstock

NO_3_^−^ serves as a valuable nitrogen feedstock due to its relatively low bond dissociation energy (BDE) (204 kJ mol^−1^) and high solubility in water.^[^ ^18^ ^]^ The rising presence of NO_3_^−^ in the environment, particularly in groundwater, is largely attributed to anthropogenic activities, including industrial and commercial wastewater discharges, making it an abundant nitrogen source.^[^ ^19^, ^20^ ^]^ Moreover, removing NO_3_^−^ from water sources can improve aquatic ecosystems and mitigate health risks, as this pollutant is associated with health afflictions such as methemoglobinemia.^[^ ^21^, ^22^ ^]^ This section reviews advanced catalysts and strategies for C—N coupling reactions using CO_2_ and NO_3_^−^ (**Table** **1**, R4).^[^ ^23^, ^24^ ^]^ The catalysts can be divided into three categories based on the respective design strategy: Cu‐based, alternative transition metals, and defect‐engineered systems.

(R4) $$2{\text{NO}}_{3}^{-}+{\text{CO}}_{2}+16e^{-}+18H^{+}\to \text{CO}{\left({\text{NH}}_{2}\right)}_{2}+7H_{2}O$$

**Table 1:** Reported electrocatalysts for C—N coupling in urea synthesis using CO_2_ and NO_3_^−^ as reactant sources.

Catalyst	Electrolyte	Electrochemical cell	*J*_urea_ [mA cm^−2^]	Potential [V_RHE_]	FE [%]	Yield	Stability	Detection method	References
V_o_–InOOH	0.1 M KNO_3_	H‐cell	0.36	−0.5	51	9.8 mmol g^−1^ h^−1^	10	DAMO‐TSC	[44]
Fe–Fe_3_O_4_/CNs	0.1 M KNO_3_	H‐cell	0.72	−0.65	17	24.2 mmol g^−1^ h^−1^	10	Urease method	[39]
Ru–Cu CF	0.1 M KNO_3_	Single cell	2.54	0.13	25	151.6 μg h^−1^ mg_cat_^−1^	12	DAMO‐TSC	[138]
In(OH)_3_–S	0.1 M KNO_3_	H‐cell	0.53	−0.6	53	8.89 mmol g^−1^ h^−1^	8	DAMO‐TSC	[42]
TiO_2_/Nafion	0.1 M KNO_3_	H‐cell	–	−0.5	40	N/A	2	Urease method	[52]
Cu/ZnO GDEs	0.1 M KNO_3_	Flow cell	1.12	−0.3	37	16 mmol g^−1^ h^−1^	5	DAMO‐TSC	[28]
CuWO_4_	0.1 M KNO_3_	H‐cell	0.95	−0.2	70	1.64 mmol g^−1^ h^−1^	10	Urease method	[18]
FeNi_3_	0.1 M KNO_3_	H‐cell	–	−0.9	17	8.23 mmol g^−1^ h^−1^	–	Urease method	[139]
Co_1_–TiO_2_	0.1 M KNO_3_	H‐cell	18.4	−0.8	36	212.8 mmol g^−1^ h^−1^	–	Urease method	[140]
XC72R–AuPd	0.025 M KNO_3_ + 0.075 M KHCO_3_	H‐cell	1.4	−0.5	15.6	3.4 mmol g^−1^ h^−1^	10	DAMO‐TSC	[62]
Cu–GS‐800	0.1 M KNO_3_ + 0.1 M KHCO_3_	H‐cell	7.56	−0.9	28	30.63 mmol g^−1^ h^−1^	–	DAMO‐TSC	[63]
F‐CNT‐300	0.1 M KNO_3_ + 0.1 M KHCO_3_	H‐cell	0.30	−0.65	18	6.36 mmol g^−1^ h^−1^	8	DAMO‐TSC	[141]
Bi:10%In/C NPs	0.1 M KNO_3_ + 0.1 M KHCO_3_	H‐cell	–	−0.45	20	10.1 mmol g^−1^ h^−1^	–	Urease method	[142]
m‐Cu_2_O	10 mM KNO_3_ + 0.1 M KHCO_3_	H‐cell	1.97	−1.3	9	29.2 mmol g^−1^ h^−1^	–	Urease method	[143]
6 Å Cu	0.1 M KNO_3_ + 1.0 M KOH	Flow cell	115.25	−0.41	51.7	7541.9 37 μg h^−1^ mg_cat_^−1^	50	^1^H‐NMR	[27]
B–FeNi–DASC	50 mM KNO_3_ + 0.1 M KHCO_3_	H‐cell	7.57	−1.5	17.8	20.2 mmol g^−1^ h^−1^	–	Urease method	[74]
Cu_1_–CeO_2_	50 mM KNO_3_ + 0.1 M KHCO_3_	H‐cell	4.68	−1.6	NA	52.84 mmol g^−1^ h^−1^	4	Urease method	[144]
V_o_–CeO_2_‐750	50 mM KNO_3_ + 0.1 M KHCO_3_	H‐cell	1.52	−1.6	NA	15.7 mmol g^−1^ h^−1^	5	Urease method	[45]
3D Zn/Cu hybrid	1000 ppm KNO_3_ + 0.1 M KHCO_3_	Flow cell	10.00	−0.8	75	55.3 mmol g^−1^ h^−1^	32	DAMO‐TSC	[145]
Cu/Zn	0.1 M KNO_3_ + 0.2 M KHCO_3_	H‐cell	3.13	−1.02	9	0.00729 mmol cm^−2^ h^−1^	12	^1^H‐NMR	[29]
CuInS_2_/TF	0.05 M KNO_3_ + 0.05 M KHCO_3_	H‐cell	–	−0.8	20	50.29 mmol g^−1^ h^−1^	–	DAMO‐TSC	[146]
CuO_50_ZnO_50_	0.1 M Na_2_NO_3_ + 0.1 M Na_2_SO_4_	H‐cell	–	−0.8	41	NA	–	Urease method	[147]
Cu–Bi heterostructure	0.1 M KNO_3_ + 0.2 M KHCO_3_	H‐cell	–	−0.6	23.5	80.3 μg h^−1^ mg_cat_^−1^	10	Urease method	[148]
a–SnBi NS/rGO	0.1 M KHCO_3_	Flow cell	–	−0.4	78.36	462.37 μg h^−1^ mg_cat_^−1^	10	Urease method	[149]
SrCo_0.39_Ru_0.61_O_3−δ_	0.1 M KNO_3_	H‐cell	–	−0.7	34.1	1522 μg h^−1^ mg_cat_^−1^	360	DAMO‐TSC	[150]

### 2.2 Cu‐based Systems

**Figure 2:**
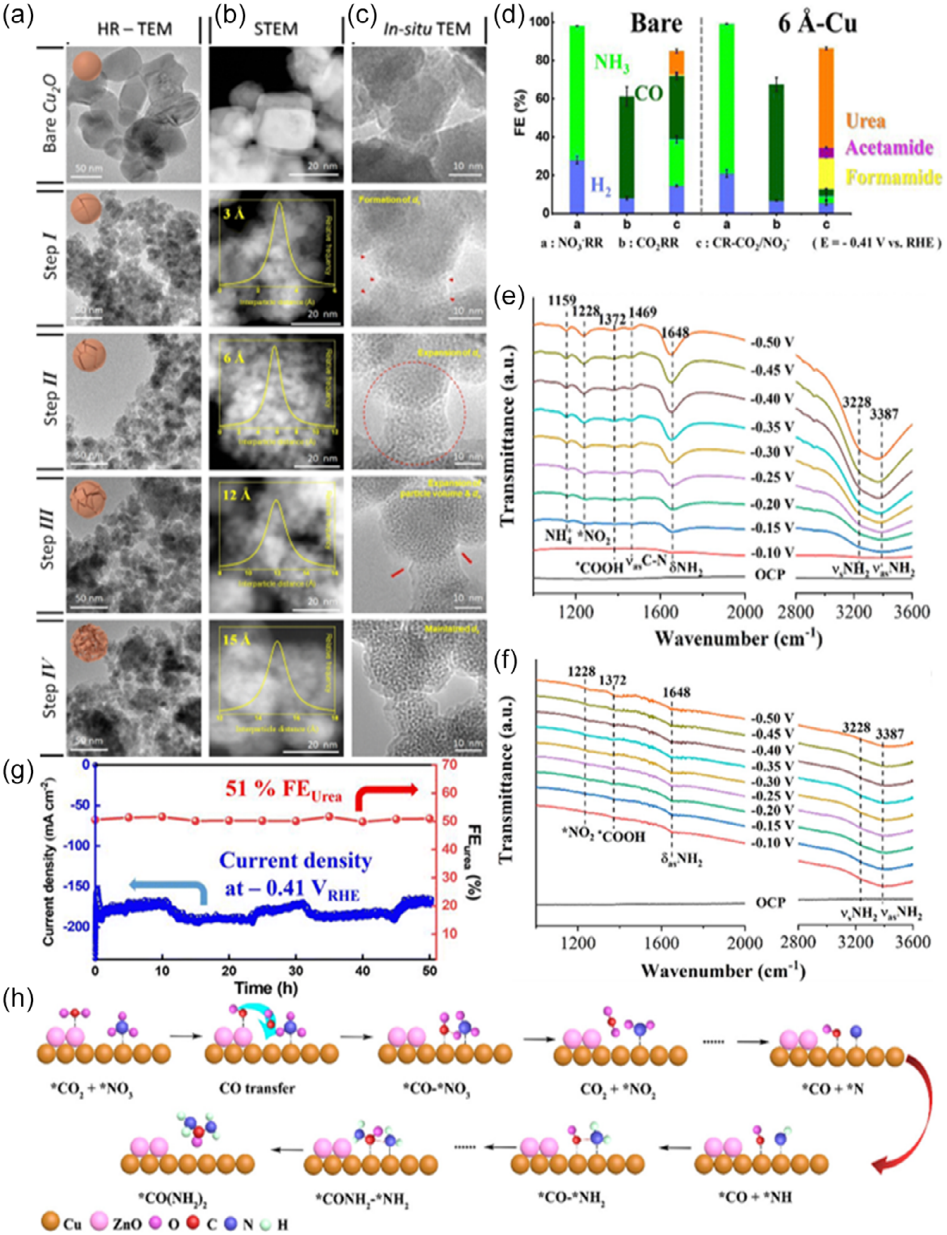
Electron microscopic analysis of bare and lithiated Cu_2_O according to a degree of lithiation. a) Ex situ HR‐TEM images with their representative scheme. Structure and morphology evolution were described with the scheme on HR‐TEM images. b) STEM images of Cu_2_O with *d*_s_. The generated *d*_s_ were observed from the HAADF‐STEM image. The distribution of *d*_s_ is described in each STEM image as an inset graph. c) Time‐lapse TEM images along lithiation progress were monitored. Structural changes of Cu_2_O during lithiation were investigated in real time at 0.5 V bias by a TEM. d) The product analysis of NO_3_^−^RR, CO_2_RR, and CR‐CO_2_/NO_3_^−^ on bare and 6 Å‐Cu at −0.41 V_RHE_. Reproduced with permission.^[^ ^27^ ^]^ Copyright 2023, Royal Society of Chemistry. In situ ATR‐FTIR spectra of e) Cu_1.0_/ZnO_0.5_ GDE and f) pure Cu GDE. Reproduced with permission.^[^ ^28^ ^]^ Copyright 2023, American Chemical Society. g) Stability test of CR‐CO_2_/NO_3_ using 6 Å Cu at −0.41 V_RHE_. The current density was monitored for 50 h (blue sphere) and FE_urea_ was calculated for every 5 h (red sphere). Reproduced with permission.^[^ ^27^ ^]^ Copyright 2023, Royal Society of Chemistry. h) Proposed mechanism of CO‐mediated NO_3_^−^ reduction for urea sythesis. Reproduced with permission.^[^ ^28^ ^]^ Copyright 2023, American Chemical Society.

Cu and Cu‐based systems are extensively studied for both the NO_3_RR and CO_2_RR due to Cu's optimal binding energies for C‐ and N‐ intermediates. This property enables researchers to leverage insights from these reactions to facilitate C—N coupling in urea synthesis.^[^ ^23^, ^24^, ^25^, ^26^ ^]^ Very recently, Shin et al. synthesized a monometallic Cu catalyst with an atomic‐scale spacing of 6 Å ± 0.11 Å between two Cu facets through the electrochemical lithiation of Cu_2_O nanoparticles. This catalyst achieved a urea Faradiac efficiency (FE) of 52% and a yield rate of 7541.9 μg h^−1^ mg^−1^ and demonstrated stability for 50 h at −0.4 V versus reversible hydrogen electrode (*V*_RHE_).^[^ ^27^ ^]^ Scanning transmission electron microscopy (STEM), high‐resolution transmission electron microscopy (HR‐TEM), and in situ TEM revealed atomic scale spacings (*ds*) in the 10 nm lithiated particles, ranging from 3 to 15 Å (**Figure** **2**a–c), achieved by controlling the degree of lithiation. Compared to pristine Cu, which achieved a FE of 15% for urea at −0.4 V_RHE_, the 6 Å Cu catalyst demonstrated an 18.8‐fold higher partial current density for urea at 115.25 mA cm^−2^ (Figure 2d,g). Notably, all tested *ds*‐Cu catalysts outperformed pristine Cu, supporting the conclusion that atomic spacing promotes C—N coupling. In situ X‐ray absorption near edge structure (XANES), Raman spectroscopy, and electrochemical surface area (ECSA) analysis confirmed that the performance increase was due to *ds*, rather than changes in oxidation state or ECSA. Density functional theory (DFT) calculations further indicated that the 6 Å spacing between Cu facets promotes a more kinetically favorable C—N coupling reaction, as electron density from the Cu surface stabilizes reaction intermediates.

**Figure 3:**
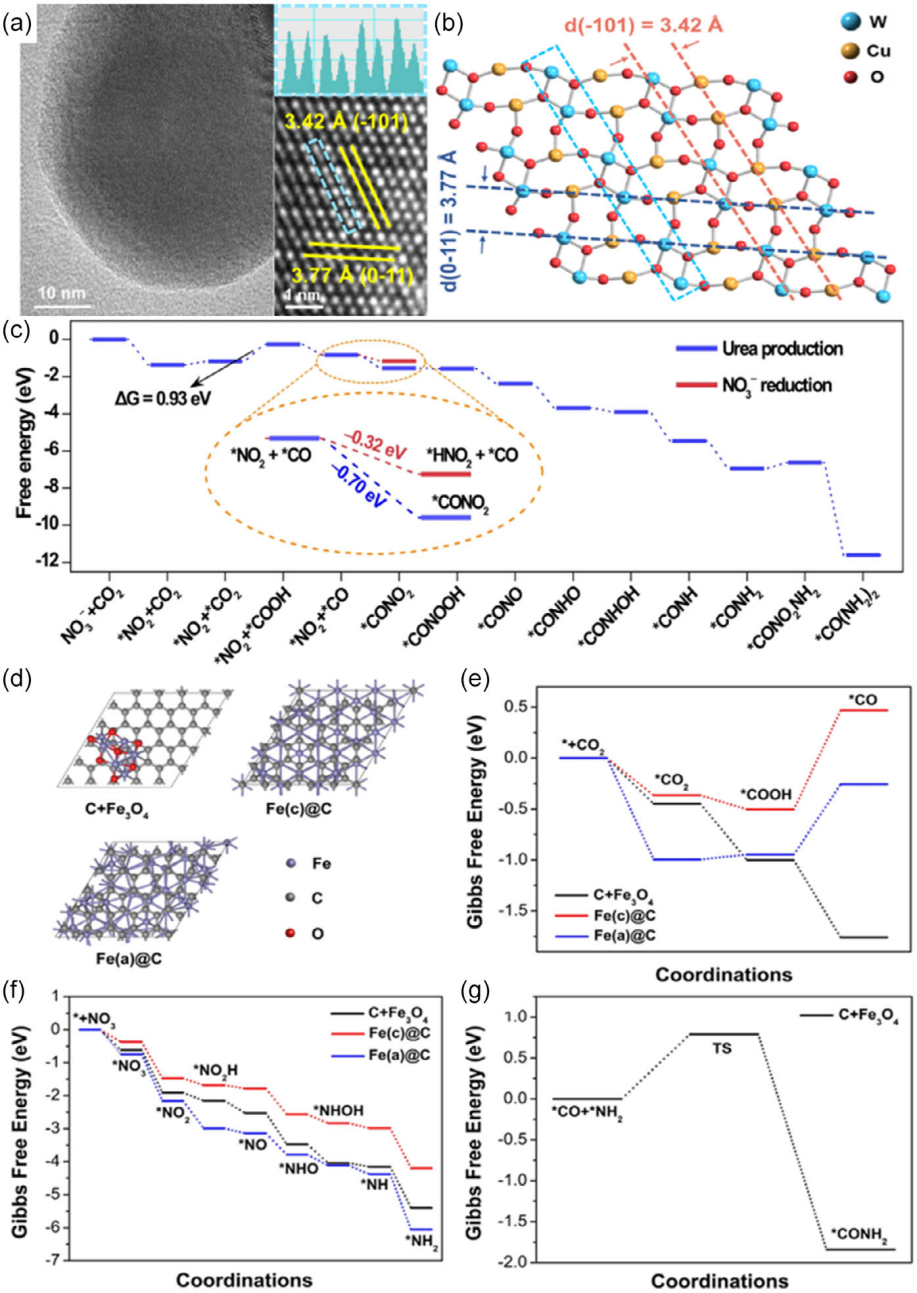
a) TEM and atomic‐resolution HAADF‐STEM image with line profile of the corresponding area. b) Atomic structure of CuWO_4_ (111) facet. c) Free‐energy diagram for urea production and NO_3_^−^ reduction on the CuWO_4_ (111) facet. Reproduced with permission.^[^ ^18^ ^]^ Copyright 2023, Springer Nature. d) Structures of C + Fe_3_O_4_, Fe(c)@C and Fe(a)@C. e) Free energy profiles of CO_2_ reduction to *CO on C + Fe_3_O_4_, Fe(c)@C and Fe(a)@C. f) Free energy profiles of NO_3_^−^ reduction to *NH_2_ on C + Fe_3_O_4_, Fe(c)@C and Fe(a)@C. g) Free energy profile of C–N coupling on C + Fe_3_O_4_. Reproduced with permission.^[^ ^39^ ^]^ Copyright 2023, Wiley‐VCH.

Beyond monometallic Cu, bimetallic Cu catalysts have also shown promise in promoting C—N coupling for urea synthesis.^[^ ^18^, ^28^, ^29^ ^]^ The combination of Cu with another metal enables a balance in the adsorption and surface coverage of *CO and nitrogen intermediate (*NH_2_, *NO_2_, *NO), facilitating more favorable reaction kinetics. This balance is crucial, as the coupling between these intermediates is considered a rate‐determining step.^[^ ^28^, ^29^, ^30^ ^]^ This design strategy was exemplified by Wang et al. who utilized a stacked tandem layer of a Cu_1.0_/ZnO_0.5_ gas diffusion layer (GDL) to achieve a urea FE of 37% and a yield rate of 3.2 μmol h^−1^ cm^−2^ at −0.3 V_RHE_.^[^ ^28^ ^]^ The ZnO catalyst layer supplied a concentrated flow of CO, ensuring high surface coverage, while Cu generated *NH_2_ intermediates, thus facilitating the C—N coupling reaction toward urea. Attenuated Fourier transform infrared spectroscopy revealed a *COOH intermediate vibration band appearing at 1372 cm^−1^ (Figure 2e,f). It was determined that *COOH generation enables efficient hydrogenation and reduction of the identified *NO_*x*_ and *NH_2_ intermediates, thereby promoting C—N coupling to urea—a finding consistent with other reports (Figure 2h).^[^ ^28^ ^]^ To tailor the adsorption of N intermediates, Li et al. developed an alternating bimetallic CuWO_4_ catalyst for efficient C—N coupling to urea, drawing inspiration from the high‐valence Mo^4+^ dinucleotide in cyanobacteria, which facilitates the reduction of NO_3_^−^ to *NO_2_.^[^ ^31^, ^32^ ^]^ While WO_3_ is effective at reducing NO_3_^−^ to *NO_2_, Cu excels in the efficient CO_2_ reduction to CO, making CuWO_4_ a well‐balanced catalyst for this application.^[^ ^18^, ^33^ ^]^ High‐angle annular dark‐field scanning transmission electron microscopy (HAADF‐STEM) image confirmed the disruption of continuous Cu ensembles through WO_4_ domains in CuWO_4_ (**Figure** **3**a). Additionally, Cu is known to promote C—C coupling, leading to other carbon byproducts. Therefore, separating Cu atoms by WO_4_ within the catalyst structure is crucial for suppressing the formation of undesired CO_2_RR products (Figure 3b).^[^ ^34^, ^35^ ^]^ It was elucidated that the electron transfer from the carbon of *CO to the nitrogen of *NO_2_ has a lower free energy (−0.70 eV), making C—N coupling more favorable compared to the *CO to *NO_2_H intermediate pathway (−0.32 eV). This finding suggests a likely pathway through *CO and *NO_2_ coupling (Figure 3c).^[^ ^18^ ^]^ This mechanism also played a crucial role in minimizing byproduct formation, as *NO_2_ became less available for further hydrogenation to NH_3_ after C—N coupling had occurred. As a result, the CuWO_4_ catalysts achieved an FE of 70% and a yield rate of 98.5 μgh^−1^ mg^−1^ at −0.2 V_RHE_.^[^ ^18^ ^]^

### 2.3 Alternative Transition Metal‐based Systems

Beyond Cu, other transition metal catalysts, particularly Fe‐based systems, have been investigated. Inspired by the widespread use of Fe‐based catalysts in the HB process and their performance in CO_2_RR and NO_3_RR, researchers have also explored their potential in the C—N coupling reaction.^[^ ^36^, ^37^, ^38^ ^]^ Geng et al. reported an FE of 16.5 ± 6.1% and a urea yield of 1341.3 ± 112.6 μg h^−1^ mg_cat_^−1^ using carbon‐encapsulated amorphous iron and iron oxide nanoparticles on carbon nanotubes (Fe(a)@C–Fe_3_O_4_/CNTs).^[^ ^39^ ^]^

Charge density difference analysis compared the p orbitals of carbon encapsulated on crystalline iron (Fe(c)@C), revealing that the graphic C in Fe(a)@C enhanced the adsorption and activation of CO_2_ and NO_3_^−^.^[^ ^39^ ^]^ Furthermore, Fe(a)@C and Fe_3_O_4_ acted as dual adsorption sites, with Fe(a)@C producing a *NH_2_ intermediate and Fe_3_O_4_ generating a *CO intermediate, thereby facilitating C—N coupling (Figure 3e–g).^[^ ^39^ ^]^ Notably, the coupling species involved are *CO and *NH_2_, as opposed to *CO and *NO_2_ reported in other studies.^[^ ^18^ ^]^

### 2.4 Defect‐Engineered Systems

**Figure 4:**
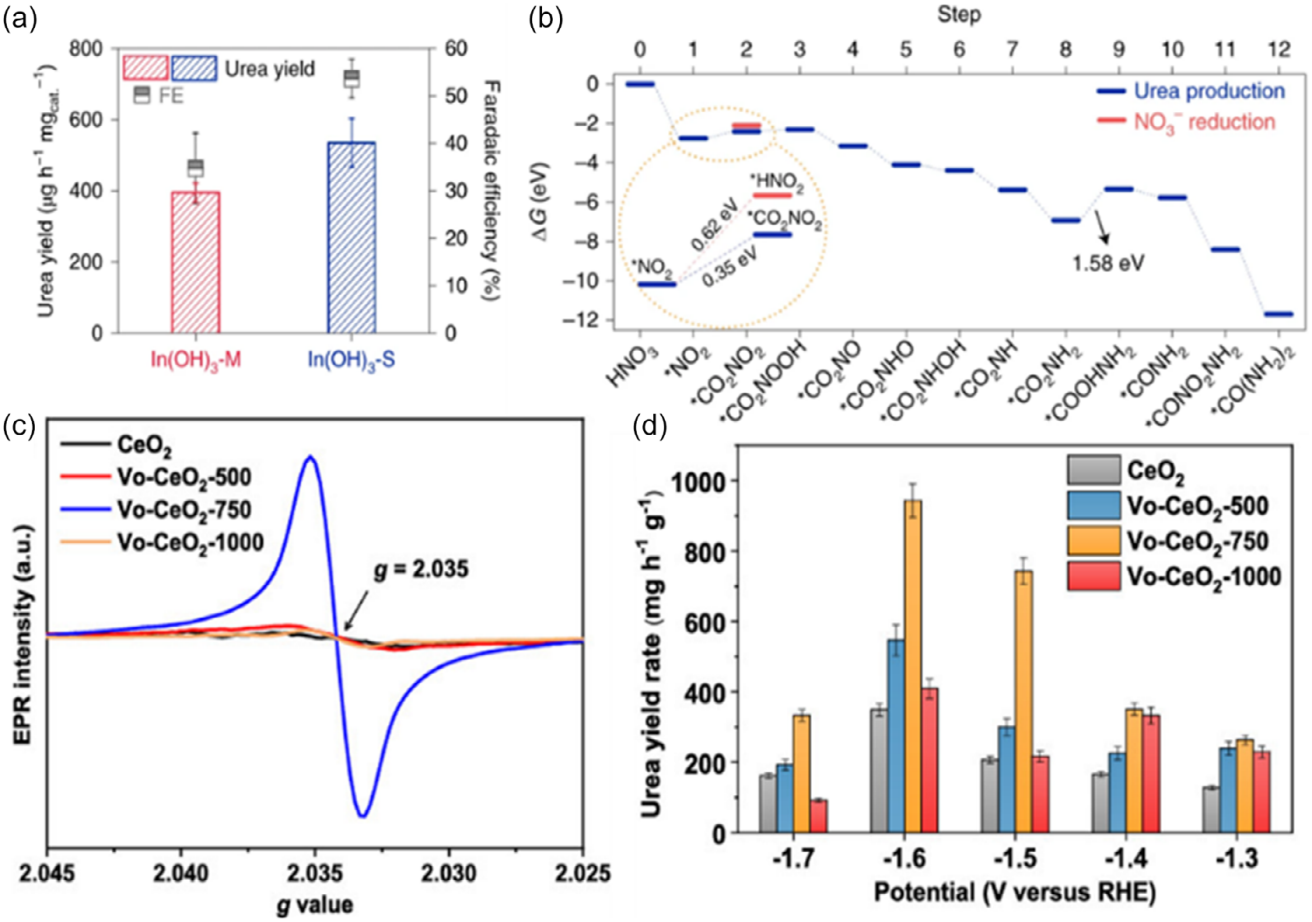
a) Urea synthesis performance comparison between In(OH)_3_–S and In(OH)_3_–M at −0.6 V_RHE_. b) Free‐energy diagram for urea production on the {100} facets of In(OH)_3_ at 0 V versus RHE. Reproduced with permission.^[^ ^42^ ^]^ Copyright 2021, Springer Nature. c) EPR spectra of CeO_2_, V_o_–CeO_2_–500, V_o_–CeO_2_–750, and V_o_–CeO_2_–1000. d) Urea yield rates of CeO_2_, V_o_–CeO_2_–500, V_o_–CeO_2_–750, and V_o_–CeO_2_–1000 at various applied potentials. Reproduced with permission.^[^ ^45^ ^]^ Copyright 2022, American Chemical Society.

Facet engineering has emerged as a promising design strategy to enhance electrocatalytic activity.^[^ ^40^, ^41^ ^]^ This concept is supported by the work of Lv et al. which demonstrated that indium hydroxide (In(OH)_3_) can promote a preferential pathway for C—N coupling on specific facets.^[^ ^42^ ^]^ The performance of the In(OH)_3_–S (In(OH)_3_ with single {100} facets) electrocatalyst with only a (100) facet was compared to that of In(OH)_3_–M with both (110) and (100) facets. At −0.6 V_RHE_ In(OH)_3_–M (In(OH)_3_ with mixed facets of {100} and {110}) achieved an FE of 35% and a yield of 392.6 mg h^−1^ g^−1^, while In(OH)_3_–S reached an FE of 53% and a yield of 533.1 mg h^−1^ g^−1^(**Figure** **4**a). The presence of OH groups between the (110) facets is believed to weaken the adsorption energy of *NO_2_ of −0.52 eV, compared to −2.48 eV on the (100) facet, thereby hindering urea formation. Additionally, the adjacent In atoms on the (100) facet facilitate the adsorption of the *CO_2_ intermediate through an In—O—C—O—In configuration. The initial C—N coupling step between *CO_2_ and *NO_2_ occurs at a lower energy barrier 0.35 eV, compared to the protonation of *NO_2_ to *HNO_2_, which has a barrier of 0.62 eV (Figure 4b). Additionally, CO_2_ saturation induces an electron capture effect, generating a surface hole accumulation layer. As a result, protons are repelled, inhibiting HER.^[^ ^42^, ^43^ ^]^ The introduction of oxygen vacancies (V_o_) of non‐Cu‐based catalysts has shown promise for enhancing C—N coupling in urea generation.^[^ ^44^, ^45^ ^]^ This approach allows modification of the electronic surface structure, enhancing chemisorption of CO_2_ and NO_3_^−^, and optimizing conditions for subsequent reactions.^[^ ^46^ ^]^ Lv et al. demonstrated this with an InOOH catalyst, where V_o_ was introduced via a solvothermal method, yielding V_o_–InOOH as confirmed by electron paramagnetic resonance (EPR).^[^ ^44^ ^]^ The V_o_ provided additional surface electrons to activate and adsorb CO_2_, effectively inhibiting the HER. Furthermore, V_o_–InOOH outperformed pristine InOOH, with both the FE and yield of urea increasing from 26.3 to 51% and 378.4 to 592.5 μg h^−1^ mg_cat_^−1^ at −0.5 V_RHE_.^[^ ^44^ ^]^ A similar strategy was applied to CeO_2_ nanorods, where Wei et al. introduced varying levels of V_o_ by controlling annealing temperatures.^[^ ^45^ ^]^ Increasing the annealing temperature led to a higher concentration of V_o_, as demonstrated by EPR. V_o_–CeO_2_–750 exhibited the strongest signal at *g* = 2.035, with sintering occurring at higher annealing temperatures (Figure 4c). At −1.6 V_RHE_, V_o_–CeO_2_–750 exhibited a yield rate three times higher than pristine CeO_2_, reaching 943.6 mg h^−1^ g^−1^ (Figure 4d).^[^ ^45^ ^]^ The coupling likely proceeds through *CO and *NO intermediates, with the V_o_ sites stabilizing *NO and lowering the coupling reaction barrier compared to pristine CeO_2_.^[^ ^45^ ^]^

### 2.5 NO_2_^−^ as the Nitrogen Source

Like NO_3_^−^, NO_2_^−^ can also serve as a nitrogen source due to its lower BDE (305 kJ mol^−1^) compared to N_2_ and its high solubility in water.^[^ ^47^ ^]^ In fact, NO_2_^−^ is a common byproduct in electrocatalytic C—N coupling reactions involving CO_2_ and NO_3_^−^ reactants.^[^ ^18^, ^29^ ^]^ This process involves 12 electron and 14 proton transfers, and Cu‐based catalysts have shown promise in facilitating these reactions (**Table** **2**) (R5).^[^ ^22^ ^]^

(R5) $$2{\text{NO}}_{2}^{-}+{\text{CO}}_{2}+12e^{-}+14H^{+}\to \text{CO}{\left({\text{NH}}_{2}\right)}_{2}+5{\text{ H}}_{2}O$$

**Table 2:** Reported electrocatalysts for C—N coupling in urea synthesis using CO_2_ and NO_2_^−^ as reactant source.

Catalyst	Electrolyte	Electrochemical cell	*J*_urea_ [mA cm^−2^]	Potential [V_RHE_]	FE [%]	Yield	Stability	Detection method	References
ZnO–V	0.2 M NaHCO_3_ + 0.1 M NaNO_2_	H‐cell	–	−0.79	23.26	16 mmol g_cat_^−1^ h^−1^	15	DAMO‐TSC	[89]
Cu–TiO_2_–V_o_	0.2 M KHCO_3_ + 0.02 M KNO_2_	H‐cell	20.8	−0.40	43.1	20.8 mol h^−1^ s^−1^	–	Urease method	[49]
Te–Pd NCs	0.1 M KHCO_3_ + 0.01 M KNO_2_	H‐cell	0.1	−1.10	12.2	–	5	DAMO‐TSC	[151]
AuCu SANFs	0.5 M KHCO_3_ + 0.01 M KNO_2_	H‐cell	0.49	−1.05	24.7	64.8 mmol g_cat_^−1^ h^−1^	–	DAMO‐TSC	[50]
Co–NiO*x* @GDY	0.01 M NaNO_2_	H‐cell	3.86	−0.7	64.3	15.2 mmol g_cat_^−1^ h^−1^	–	DAMO‐TSC	[152]

**Figure 5:**
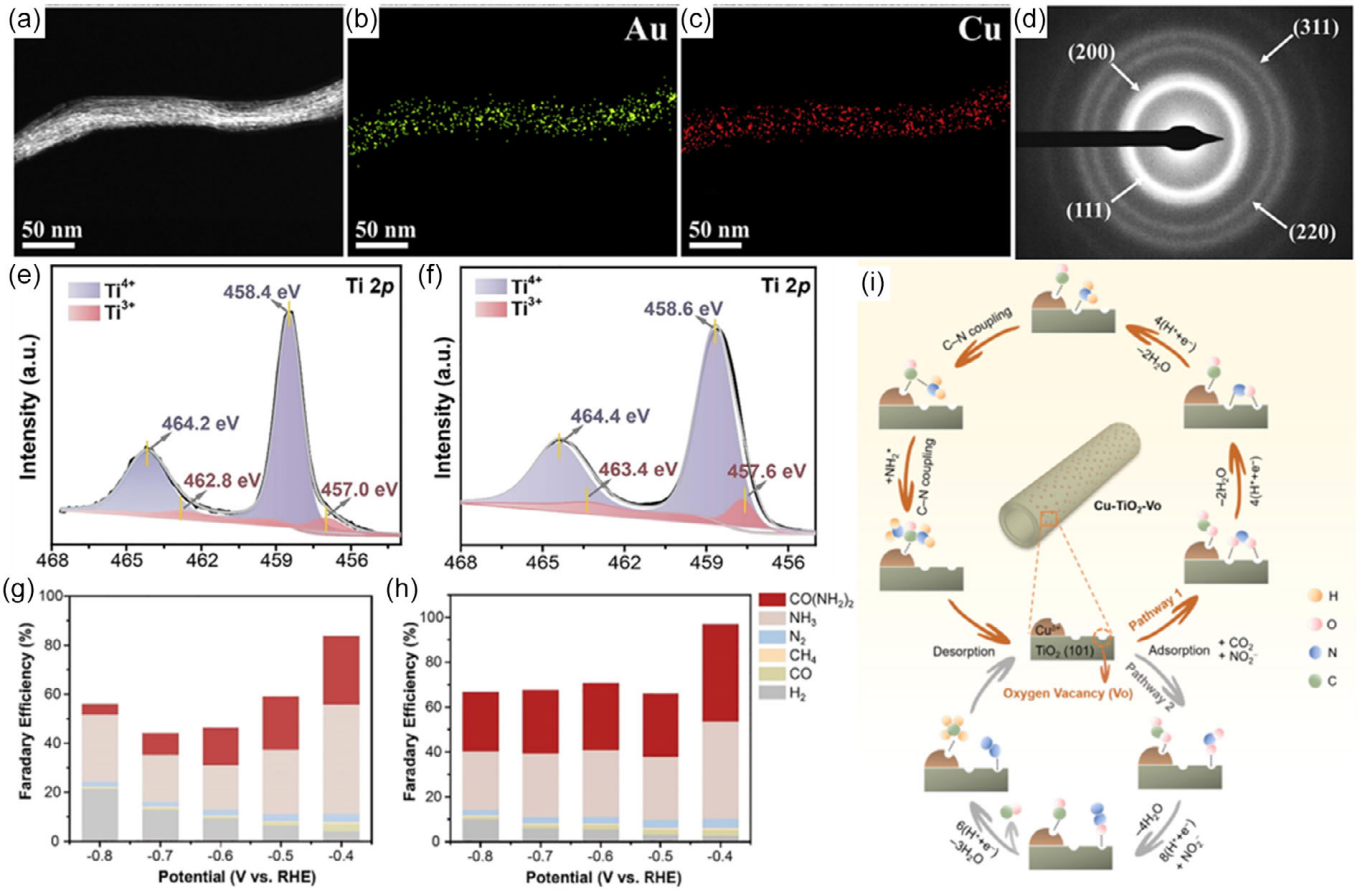
a) HAADF‐STEM image and b,c) energy‐dispersive X‐ray spectroscopy mapping images of the AuCu SANFs. d) SAED image of the AuCu SANFs. Reproduced with permission.^[^ ^50^ ^]^ Copyright 2022, Elsevier. Ti 2*p* XPS spectra of e) undoped TiO_2_ and f) Cu–TiO_2_. Faradaic efficiencies of major reduction products for g) undoped TiO_2_ and h) Cu–TiO_2_. i) Possible synthesis mechanisms of urea and other byproducts for coreduction of CO_2_ and NO_2_^−^ on Cu–TiO_2_. NO_2_^−^ ions are adsorbed on the bi‐Ti^3+^ active sites in a side‐on manner (Pathway 1) or an end‐on manner (Pathway 2). Reproduced with permission.^[^ ^49^ ^]^ Copyright 2020, Elsevier.

The first report on C—N coupling for urea synthesis from NO_2_^−^ and CO_2_ was published by Shibata et al. in 1995, using a Cu‐GDL achieving an FE of 10% versus −1.5 V_RHE_.^[^ ^48^ ^]^ Since then, performance has improved through strategies such as the introduction of V_o_ and synergistic alloy effects.^[^ ^49^ ^]^ For example, Liu et al. synthesized 1D AuCu self‐assembled nanofibers (SANFs), achieving high urea yield rate of 64.8 mmol g_cat_^−1^ h^−1^ with an FE of 24.7% at −1.05 V_RHE_.^[^ ^50^ ^]^ This performance was attributed to the bimetallic synergistic effects and the presence of the Boerdijk–Coxeter‐type helices dominated by (111) facets, confirmed by selected area electron diffraction (SAED), which introduced surface defects (**Figure** **5**a–d).^[^ ^50^, ^51^ ^]^ These electronic modifications increased the availability of active sites, facilitating C—N coupling for urea synthesis via a *CO and *NH_2_ intermediate pathway. Alternatively, Cao et al. significantly increased the Vo concentration in TiO_2_ by doping Cu onto anatase TiO_2_ nanotubes via a wet chemical method, as confirmed by XPS (Figure 5e,f).^[^ ^49^, ^52^ ^]^ Doping TiO_2_ with Cu increased the FE and urea yield rate to 43.1% and 20.8 mol h^−1^ s^−1^, compared to undoped TiO_2_ at 27.3% and 5.91 mol h^−1^ s^−1^ at −0.4 V_RHE_ (Figure 5g,h).^[^ ^49^ ^]^ The Cu dopants enhanced CO_2_ adsorption, favoring reduction to the *CO intermediate, while the V_o_‐rich TiO_2_ facilitated NO_2_^−^ activation to the *NH_2_ intermediate, optimizing C—N coupling (Figure 5i).

### 2.6 NO as the Nitrogen Source

NO is often regarded as a key intermediate in NO_3_^−^ and NO_2_^−^ reductions. Consequently, NO with a BDE of 631 kJ mol^−1^ has also been explored as a nitrogen source in C—N coupling (R6).^[^ ^53^, ^54^, ^55^ ^]^ A limitation of using NO as a nitrogen source is its relatively low environmental availability, with an annual mean concentration of 20–90 μg m^−3^, making it less accessible than other nitrogen feedstocks despite its status as an air pollutant.^[^ ^56^ ^]^

(R6) $$2NO+{\text{CO}}_{2}+10e^{-}+10H^{+}\to \text{CO}{\left({\text{NH}}_{2}\right)}_{2}+3H_{2}O$$

The first report of C—N coupling for urea synthesis from NO and CO_2_ was likely published in 2022, where 10 metal catalysts were screened, with Zn foil showing the highest urea yield rate at −0.92 V_RHE_.^[^ ^57^ ^]^

ZnO nanosheets were deposited onto Zn foil, which underwent successive electroreduction to form ZnO nanobelts (NB). In 0.2 M potassium bicarbonate (KHCO_3_) with a NO to CO_2_ molar ratio of 3:7, the ZnO NBs achieved a urea FE of 11.26% at −0.92 V_RHE_. Online differential electrochemical mass spectrometry (DEMS), in situ ATR‐FTIR, and DFT calculations suggested that the reaction proceeds via *CO and *NH surface intermediate species, with the C—N coupling band appearing on the IR spectrum at 1465 cm^−1^.^[^ ^57^ ^]^

### 2.7 N_2_ as the Nitrogen Source

N_2_, which makes up 78% of the atmosphere, is a widely available and economically feasible nitrogen feedstock.^[^ ^58^ ^]^ However, its linear structure, poor water solubility, and strong BDE (941 kJ mol^−1^) make it more challenging to activate compared to other nitrogen species previously discussed.^[^ ^59^ ^]^ Nonetheless, researchers have explored C—N coupling for urea generation from N_2_ and CO_2_ (R7) (**Table** **3**).

(R7) $$N_{2}+{\text{CO}}_{2}+6e^{-}+6H^{+}\to \text{CO}{\left({\text{NH}}_{2}\right)}_{2}+H_{2}O$$

**Table 3:** Reported electrocatalysts for C—N coupling in urea synthesis using CO_2_ and N_2_ as the reactant sources.

Catalyst	Electrolyte	Electrochemical cell	*J*_urea_ [mA cm^−2^]	Potential [V_RHE_]	FE [%]	Yield	Stability [h]	Detection method	References
PdCu/TiO_2_‐400	1.0 M KHCO_3_	Flow cell	0.16	−0.4	8.92	3.36 mmol h^−1^ g^−1^	12	Urease method	[61]
Cu–Bi	0.1 M KHCO_3_	H‐cell	–	−0.4	8.7	0.45 mg L^−1^	2	DAMO‐TSC	[64]
BiFeO_3_/BiVO_4_	0.1 M KHCO_3_	H‐cell	0.17	−0.4	17	4.94 mmol h^−1^ g^−1^	10	DAMO‐TSC	[66]
BiVO_4_	0.1 M KHCO_3_	H‐cell	0.23	−0.4	12.55	5.91 mmol h^−1^ g^−1^	10	DAMO‐TSC	[67]
CuPc NTs	0.1 M KHCO_3_	H‐cell	0.12	−0.6	12.99	143.47 μg h^−1^ mg^−1^	10	DAMO‐TSC	[153]
Ni(BO_3_)_2_–150	0.1 M KHCO_3_	H‐cell	0.31	−0.5	12.99	9.70 mmol h^−1^ g^−1^	20	DAMO‐TSC	[65]
V_N_–Cu_3_N–300	0.1 M KHCO_3_	H‐cell	0.11	−0.4	28.7	81 μg h^−1^ cm^−1^	10	DAMO‐TSC	[154]
Co‐based MOF	0.1 M KHCO_3_	H‐cell	0.49	−0.5	48.97	14.5 mmol h^−1^ g^−1^	20	DAMO‐TSC	[68]
InOOH	0.1 M KHCO_3_	H‐cell	0.25	−0.4	21	6.9 mmol h^−1^ g^−1^	20	DAMO‐TSC	[155]
MoP‐(101)	0.1 M KHCO_3_	H‐cell	0.05	−0.35	36.5	12.4 μg h^−1^ mg^−1^	5	DAMO‐TSC	[70]

**Figure 6:**
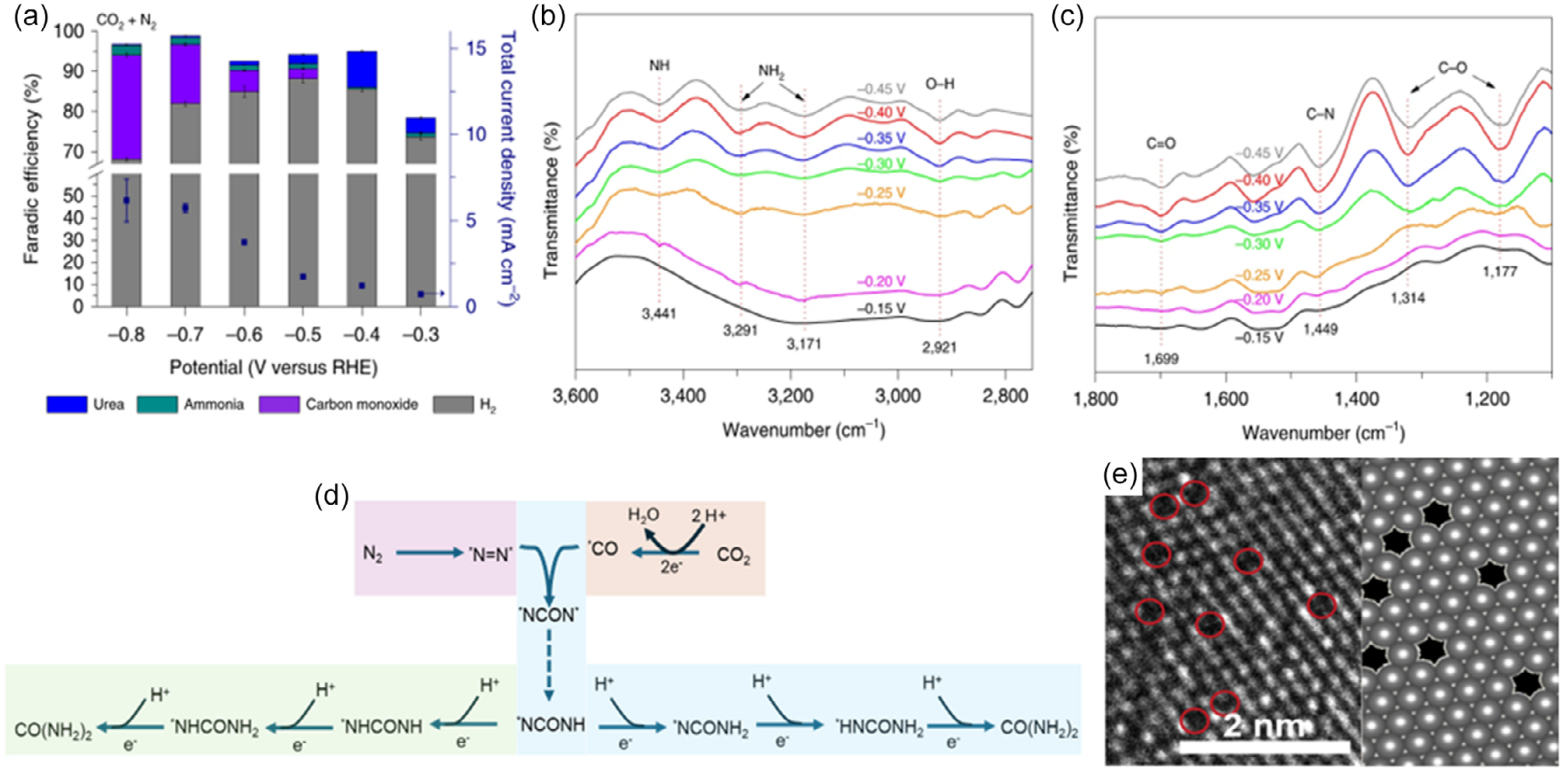
a) Faradic efficiency and the total current densities for all products at various potentials. Infrared signal b) in the range of 2750–3600 cm^−1^ and c) in the range of 1100–1800 cm^−1^under various potentials for Pd_1_Cu_1_/TiO_2_‐400 during the electrocoupling of N_2_ and CO_2_. d) reaction pathway towards urea from N_2_ and CO_2_ via alternative (blue) and distal (green) pathways. Reproduced with permission.^[^ ^61^ ^]^ Copyright 2020, Springer Nature. e) TEM image from a double‐corrected spherical aberration electron microscope. Reproduced with permission.^[^ ^64^ ^]^ Copyright 2022, Elsevier.

In 2016, Kayan and Köleli first reported C—N coupling for urea synthesis from N_2_ and CO_2_, achieving a 7% urea FE with a polypyrrole‐coated Pt electrocatalyst.^[^ ^60^ ^]^ Chen et al. slightly improved this process using PdCu alloy nanoparticles supported on TiO_2_ sheets.^[^ ^61^ ^]^ Similar to other nitrogen sources, various concentrations of V_o_ were introduced into pristine TiO_2_ nanosheets through chemical reduction followed by thermal treatment.^[^ ^49^, ^62^, ^63^ ^]^ EPR analysis revealed that an annealing temperature of 400 °C produced the highest concentration of V_o_. PdCu nanoparticles were then anchored onto the TiO_2_‐400 nanosheets to create the Pd_1_Cu_1_/TiO_2_‐400 catalyst. In a N_2_ and CO_2_ saturated 0.1 M KHCO_3_ electrolyte, this catalyst achieved an FE of 8.92% and a yield rate of 3.36 mmol g^−1^ h^−1^ at −0.4 V_RHE_ (**Figure** **6**a).^[^ ^61^ ^]^ Synchrotron radiation‐based Fourier‐transform infrared spectroscopy (SR‐FTIR) was performed to elucidate the C—N coupling mechanistic pathway.

Vibration bands for *NH_2_, *NH, C=O, and *C—O were observed, with the crucial C—N vibration band appearing at ≈1,449 cm^−1^ at −0.3 V_RHE_. The intensity of the C—N band also peaked at −0.4 V_RHE_, consistent with the electrochemical performance results (Figure 6b,c). DFT calculations indicated that C—N coupling proceeds through the initial formation of *NNH and *CO, generating an *NCONH species. The subsequent protonation of *NCONH can occur via a distal mechanism, forming *NCONH_2_, or through an alternative pathway leading to *NHCONH, with *NCONH_2_ being 0.14 eV more stable than *NHCONH (Figure 6d).

Wu et al. provided further evidence that surface defects enhance C—N coupling from N_2_ and CO_2_ using a Cu—Bi alloy.^[^ ^64^ ^]^ Their comparison of monometallic Cu and Bi with a bimetallic Cu—Bi mixture revealed that the defective surface of the Cu—Bi alloy exhibited superior performance. The alloy consisted of ordered regions with defects, which influenced the electronic structure of the surface, thereby improving the urea synthesis performance compared to both monometallic and bimetallic species (Figure 6e). The defective Cu—Bi alloy achieved an FE of 8.7 ± 1.7% and a yield rate of 0.45 mg L^−1^ ± 0.06 mg L^−1^ at −0.4 V_RHE_ in a N_2_ and CO_2_ saturated 0.1 M KHCO_3_ electrolyte.^[^ ^64^ ^]^

**Figure 7:**
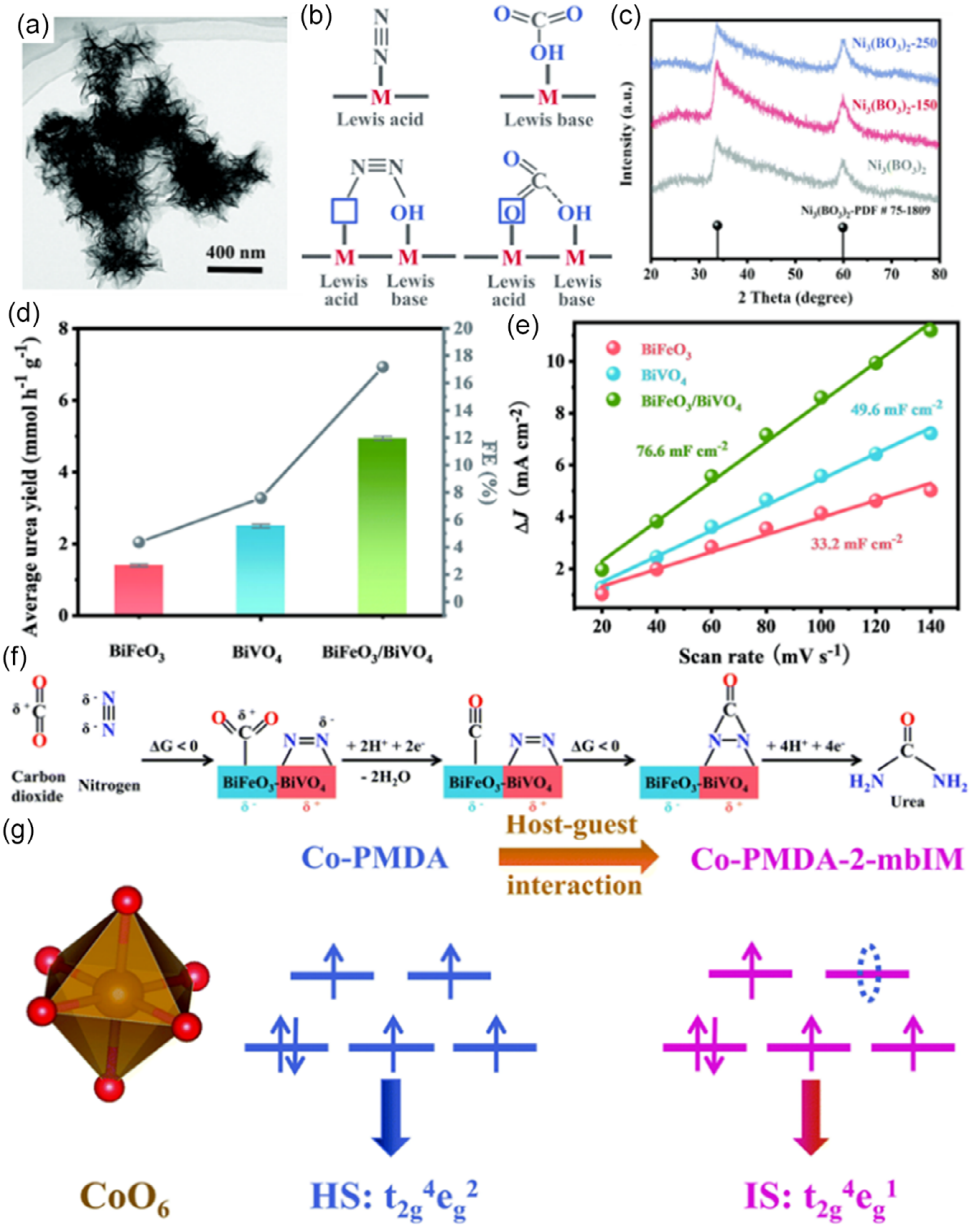
a) The TEM image of Ni_3_(BO_3_)_2_‐150 catalysts. b) Schematic illustration of the contribution of artificial frustrated Lewis pairs from adsorption. c) XRD patterns of the pristine Ni_3_(BO_3_)_2_, Ni_3_(BO_3_)_2_‐150, and Ni_3_(BO_3_)_2_‐250 catalysts. Reproduced with permission.^[^ ^65^ ^]^ Copyright 2021, Royal Society of Chemistry. d) Average urea yield and e) Δ*J* of electrocatalysts plotted against scan rate at −0.05 V versus RHE of BiFeO_3_, BiVO_4_, and BiFeO_3_/BiVO_4_. f) The schematic electrocatalytic urea production mechanism based on BiFeO_3_/BiVO_4_ p–n heterostructure synergistic effects. Reproduced with permission.^[^ ^66^ ^]^ Copyright 2021, Royal Society of Chemistry. g) A schematic illustration of spin‐state regulation in Co‐PMDA‐2‐mbIM induced by host–guest interaction. Reproduced with permission.^[^ ^66^ ^]^ Copyright 2022, Royal Society of Chemistry.

Alternative strategies for tuning the surface electronic structure, such as frustrated Lewis pairs (FLP) and heterojunctions, have also been explored. Yuan et al. designed an FLP using a flower‐like Ni_3_(BO_3_)_2_‐150 nanocrystals, focusing on optimizing surface conditions to enhance the adsorption of species (**Figure** **7**a).^[^ ^65^ ^]^ The annealing treatment cleaved the Ni—OH bond, creating unsaturated Ni active sites that functioned as Lewis acid sites where the *π* orbitals of the reactants could donate their electrons into the empty d‐orbital of Ni. The neighboring surface hydroxyls, acting as the Lewis base, could then donate their electrons to the empty σ* orbital of the adsorbed species (Figure 7b). The Lewis acid‐base ratio was optimized by annealing at 150 and 250 °C, as excessively basic conditions favored CO_2_ activation, while overly acidic conditions favored N_2_ activation (Figure 7c). The Ni_3_(BO_3_)_2_‐150 nanocrystal emerged as the optimal acid‐base ratio configuration, achieving an FE of 20% and a yield rate of 9.70 mmol h^−1^ g_cat_^−1^ at −0.4 V_RHE_.^[^ ^65^ ^]^

**Figure 8:**
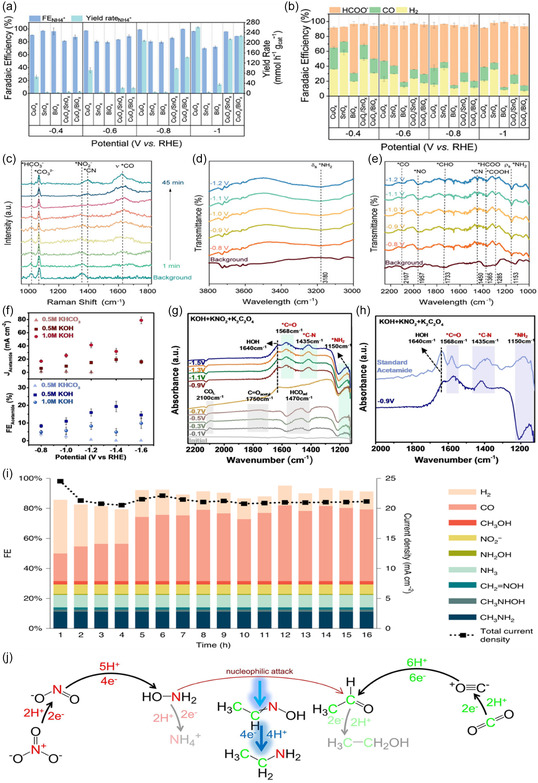
The effect of p*‐*block oxides on the CuO_*x*_ activity for formamide electrosynthesis: a) The FE and yield rate of NH_4_^+^ during NO_3_^−^ RR in Ar‐saturated 0.1 m KHCO_3_ and 0.05 m KNO_3_ solution. b) The FE_CO_ and FE_HCOO_^−^ during the CO_2_RR in CO_2_‐saturated 0.1 m KHCO_3_ solution. In situ characterization of CuO_*x*_/BiO_*x*_ catalyst: c) The in situ SR‐FTIR with enlarged wavelength windows at d) 3800–3000 cm^−^^1^ and e) 1000–2250 cm^−^^1^. The potential unit displayed in the in situ SR‐FTIR figures is in V versus RHE. All experiments were carried out in CO_2_‐saturated 0.2 m KHCO_3_ and 0.02 m KNO_2_ solution. Reproduced with permission.^[^ ^75^ ^]^ Copyright 2023, Wiley‐VCH. f) The partial current densities and FE versus the applied potential for acetamide on Cu nanoparticles during CO_2_ and nitrite coreduction in different electrolytes with various pH values. g) Potential‐dependent, attenuated total reflection infrared (ATR‐IR) spectra on Cu nanoparticles with oxalate as the C source and NO_2_^−^ as the N source in 0.5 m KOH. h) Full range spectra on Cu nanoparticles with oxalate as the C source and NO_2_^−^ as the N source in 0.5 m KOH (dark blue) and standard acetamide (light blue) at −0.9 V versus RHE. Reproduced with permission.^[^ ^81^ ^]^ Copyright 2024, Wiley‐VCH. i) Product distribution and total current density during 16 h electrolysis at −0.94 V versus RHE. The liquid products were quantified after the electrolysis was completed, and their average FE was calculated.^[^ ^16^ ^]^ j) Proposed reaction pathway to form acetaldoxime and ethylamine from electrochemical coreduction of CO2 and NO3−. The reaction intermediates shown here have been confirmed experimentally.

This strategy was further investigated by the Zhang group using BiFeO_3_/BiVO_4_ and Bi–BiVO_4_ hybrid heterostructures (**Figure** **8**d–f).^[^ ^66^, ^67^ ^]^ Compared to the pristine samples, the hybrid catalysts exhibited a higher ECSA, enhancing the exposure of active sites as well as promoting the chemisorption of N_2_ and CO_2_ by creating the space‐charge regions that promote local electrophilic and nucleophilic environments (Figure 7e). The heterojunction of BiFeO_3_/BiVO_4_ facilitated charge redistribution, optimizing N_2_ activation in the electrophilic BiVO_4_ regions. Meanwhile, adsorbed CO_2_ was reduced to CO in the nucleophilic BiFeO_3_ region. These enhanced conditions improved C—N coupling, with the *CO intermediate attacking a *N = N* intermediate to form an *NCON* intermediate. Consequently, BiFeO_3_/BiVO_4_ achieved a FE of 17.18% and a yield rate of 4.94 mmol h^−1^ g^−1^ (Figure 7d,f). Similarly, a cobalt pyromellitic dianhydride conductive metal‐organic framework (Co–PMDA–2mbIM) was developed to improve C—N coupling.^[^ ^68^ ^]^ The nucleophilic 2mbIM guest molecules facilitated interior charge transfer by bridging the local electrophilic CoO_6_ octahedron region through their lone electron pairs (Figure 7g). This induced spontaneous electron transfers promoted the N_2_ and CO_2_ adsorption and activation, with Co‐PMDA‐2mbIM achieving a yield rate of 14.47 mmol h^−1^ g^−1^ with an FE of 48.97% to urea at −0.5 V_RHE_.

Transition metal phosphides (TMP) have emerged as promising candidates due to their versatile and tunable electronic and structural properties.^[^ ^69^ ^]^ Jiao et al. reported that commercially available MoP nanoparticles achieved a urea FE of 36.5% and a yield rate of 12.4 μg h^−1^ mg^−1^ at −0.27 V_RHE_.^[^ ^70^ ^]^ DFT calculations indicated that the exposed active sites on the MoP‐(101) surface facilitated urea generation by activating CO_2_ and N_2_ through a *NCOHN intermediate.

### 2.8 Alternative C—N Coupling Products

Additional C—N products—such as formamide, acetamide, methylamine, and ethylamine—have also been observed, derived from the discussed carbon and nitrogen sources (**Table** **4**).^[^ ^16^, ^71^, ^72^ ^]^ Expanding the scope of C—N coupling beyond urea would enhance its appeal, as it could be applied to produce other valuable commodity chemicals while contributing valuable insights to the field for future advancements.

**Table 4:** Reported electrocatalysts for alternative C—N products via CO_2_ and discussed nitrogen sources.

Catalyst	Electrolyte	Electrochemical cell	*J*_product_ [mA cm^−2^]	Potential [V_RHE_]	FE [%]	Product	References
CuO_*x*_/BiO_*x*_	0.02 M KNO_2_ + 0.2 M KHCO_3_	H‐cell	125	−3.0	4.8	Formamide	[75]
Cu NPs	0.02 M KNO_2_ + 0.5 M KOH	Flow cell	20	−1.4	20	Acetamide	[81]
CuO NPs	0.1 M KNO_3_ + 0.1 M KHCO_3_	–	–	−1.0	0.36	Acetaldoxime	[71]
CuO NPs	0.1 M KNO_3_ + 0.1 M KHCO_3_	–	–	−1.0	0.30	Ethylamine	[71]
CoPc–NH_2_/CNT	0.5 M KNO_3_ + 0.1 M KHCO_3_	–	3.4	−0.92	13	Methylamine	[15]

### 2.9 Formamide

Formamide, commonly used as a feedstock for formate esters and as an ionizing solvent, is traditionally produced by reacting methyl formate with NH_3_.^[^ ^73^ ^]^ Drawing inspiration from previous Cu‐based systems for C—N coupling, Daiyan et al. reported a CuO_*x*_/BiO_*x*_ nanocomposite for electrochemical reduction of CO_2_ and NO_*x*_^−^ to formamide.^[^ ^42^, ^71^, ^74^, ^75^ ^]^ The catalyst achieved a FE of 4.8% and maximum yield rate of 134 ± 11 mmol h^−1^ g_cat_^−1^ at −3.0 V_RHE_ in a flow cell with NO_2_^−^ as the nitrogen source. P‐block metal oxides, specifically BiO_*x*_ and SnO_*x*_, have demonstrated formate production abilities from the CO_2_RR while effectively suppressing the HER.^[^ ^76^, ^77^, ^78^ ^]^ Initial systematic studies in Ar and CO_2_ saturated 0.1 M KHCO_3_ and 0.1 M KHCO_3_ with 0.05 M KNO_3_ electrolytes were probed for CO_2_RR and NO_*x*_RR activity (Figure 8a,b). The studies revealed the addition of BiO_*x*_ and SnO_*x*_ to CuO_*x*_ had no significant effect on the current density or NH_4_^+^ generation but enhanced the CO_2_RR to formate. Specifically, the FE for CuO_*x*_ increased from 52.6% to 87.2% and 65% with CuO_*x*_/SnO_*x*_ and CuO_*x*_/BiO_*x*_ at −1.0 V_RHE_. Electrolyte optimizations demonstrated that increasing the KHCO_3_ concentration to 0.5 M enhances the performance of the HER and CO_2_RR, attributed to the rise of local pH. Furthermore, replacing KNO_3_ with KNO_2_ significantly enhanced formamide yield from 8.0 ± 0.7 to 47 ± 4 mmol h^−1^ g_cat_^−1^ at −1.0 V_RHE_. This was hypothesized to be attributed to the NO_3_^−^ adsorption outcompeting CO_2_. In situ SR‐FTIR was conducted across various potentials (−0.8 to −1.2 V_RHE_), revealing the C—N vibration band at 1450 cm^−1^, with a maximum intensity at −1.0 V_RHE_ (Figure 8c–e). The appearance of *NH_2_ and *CHO bands suggests the species are key intermediates for formamide generation.

### 2.10 Acetamide

Acetamide, primarily synthesized in acidic conditions through a Beckmann rearrangement of aldoxime, has critical roles in the production of plastics and solvents.^[^ ^79^, ^80^ ^]^ Recently, Kuang et al. reported acetamide synthesis via the coreduction of CO_2_ and NO_2_^−^ with Cu NPs.^[^ ^81^ ^]^ From the reported results by Wang et al. on ethylamine, Kuang et al. performed the C—N coupling reaction in alkaline conditions to combat H_2_ generation.^[^ ^71^ ^]^ The initial CO_2_RR electrolysis demonstrated the Cu NPs produced carbon products such as CO, ethanol, and acetate. In situ ATR‐IR revealed that oxalate formation is a key intermediate for the systematic CO_2_RR study. Additionally, an NH_3_ FE of 72% was achieved during the initial NO_2_RR electrolysis. Due to the generation of (bi)carbonates via CO_2_ saturation in alkaline conditions in H‐cells and to increase catalytic performance, C—N coupling was performed via a flow cell equipped with a GDE.^[^ ^82^, ^83^ ^]^ In 0.5 M KOH, the coreduction of CO_2_ and NO_2_^−^ reached a FE of 20% at −1.4 V_RHE_ for acetamide (Figure 8f). 0.5 M KOH was found to be the optimal catholyte, as a 2% FE was observed with 0.5 M KHCO_3_ and a 5% FE in 1.0 M KOH. The decrease in FE in more alkaline conditions was attributed to the hydrolysis of acetamide to acetate. However, 1.0 M KOH was found to enhance the acetamide partial current density by 10%. ATR‐FTIR was performed with oxalate rather than CO_2_ to identify possible intermediates, which indicated a C—N band appearing at 1435 cm^−1^ at −0.9 V_RHE_ (Figure 8g,h). The results were then combined with DFT calculations and systematic studies, revealing the independent reduction of CO_2_ and NO_2_^−^ to acetaldehyde and hydroxylamine (NH_2_OH), undergoing nucleophilic addition on the catalyst surface to generate acetaldoxime. The local alkaline environment and electric field then generate acetonitrile through the dehydrogenation and dihydroxylation of acetaldoxime. Acetonitrile then undergoes hydrolysis to acetamide due to the bulk alkaline environment. Overall, this study highlights the influence of pH on the C—N coupling environment which may help to bring about future studies in the field.

### 2.11 Methylamine

Methylamine, widely used in pharmaceuticals, insecticides, and surfactants, is emerging as a promising alternative C—N product.^[^ ^84^ ^]^ Similar to the HB, the production of methylamine is performed in harsh conditions (350–450 °C, 20–40 bar) where methanol and NH_3_ are reacted over a dehydration catalyst such as a silica alumina.^[^ ^85^ ^]^ Perhaps, the first report of electrochemical methylamine synthesis from CO_2_ and NO_3_^−^ was published by Wang et al. on a Co β‐tetraaminophthalocyanine (CoPc‐NH_2_) molecular catalyst supported on CNT, with the product being detected via ^1^H‐NMR, carbon‐13 NMR, and gas chromatography–mass spectrometry.^[^ ^16^ ^]^ With a 14e^−^/15H^+^ transfer, a FE of 13% was achieved at −0.92 V_RHE_, with the catalyst remaining stable over a 16 h period (Figure 8i). CO_2_ undergoes a 4e^−^/4H^+^ transfer to generate an *OHCH, which undergoes nucleophilic attack via NH_2_OH produced via the NO_3_RR, yielding a C—N species, formaldoxime. This intermediate is then electrochemically reduced to *N‐*methylhydroxylamine, followed by the targeted methylamine. To elucidate the mechanistic pathway, systematic control experiments to identify reaction intermediates were performed. NO_2_^−^, NH_2_OH, and NO were used to elucidate the nitrogen intermediate with CO_2_. Similar experiments using CO and CH_3_OH as the carbon source were conducted, where methylamine was not observed with CH_3_OH, confirming *OHCH as the carbon intermediate. Separate DFT calculations on the CoPc‐NH_2_ catalysts were carried out by Jing et al. who deduced that the C—N coupling reaction is more favorable via desorbed HCHO and NH_2_OH species rather than adsorbed species.^[^ ^86^ ^]^

### 2.12 Ethylamine

Similar to methylamine, ethylamine has critical applications in pharmaceuticals, agriculture, and industrial processes.^[^ ^87^ ^]^ In addition to the other discussed commodity chemicals, the electrochemical synthesis of ethylamine via C—N coupling is an attractive sustainable production method when compared to the heat and pressure required on the industrial scale.^[^ ^88^ ^]^ Wang et al. reported 8 nm CuO NPs with a FE of 0.36% at −1.0 V_RHE_ toward acetaldoxime via the simultaneous reduction of CO_2_ and NO_3_^−^. With an initial electrolysis duration of 1 h, only acetaldoxime was detected via ^1^H‐NMR. When extended to 5 h, ethylamine was detected with a FE of 0.30%. An increase in H_2_ FE was also observed, attributed to the proton donation from NH_4_^+^ near the electrode surface. Acetaldoxime was identified as the key intermediate for ethylamine generation, generated via a 16e^−^/17H^+^ pathway, with a condensation reaction between acetaldehyde and NH_2_OH occurring. The subsequent cascade reduction of acetaldoxime via a 4e^−^/4H^+^ (20e^−^/21H^+^ total) then generates ethylamine (Figure 8j). Systematic control experiments for intermediate identification revealed C—N bond formation occurred from an independent acetaldehyde and NH_2_OH condensation reaction. With electrolysis of CO and acetaldehyde as the carbon source and NO_3_^−^, acetaldoxime was detected but with ethanol as the carbon source no acetaldoxime was observed, confirming the acetaldehyde pathway. Ammonium bicarbonate as the nitrogen source under CO_2_ failed to generate acetaldoxime while the product was observed with NO_2_^−^ and NH_2_OH, confirming the NH_2_OH intermediate. Additionally, the electrolysis NO_2_^−^ and NH_2_OH as the nitrogen source with CO_2_ yielded ethylamine. While catalytic performance parameters can be improved such as yield and current density, for future applications, inspiration can be taken to target future multicarbon amine products.

## 3 Detection and Quantification Methods of Urea Production

The yield of urea produced from electrochemical C—N coupling is often in the μg or μmol range and is accompanied by numerous side products from competing reactions. This makes the detection and quantification methods of paramount importance for accurately assessing the performance of catalytic systems. Currently, procedures for urea detection and quantification are typically carried out using one of the following methods: diacetylmonoxime‐thiosemicarbazide (DAMO‐TSC), urease decomposition, high‐performance liquid chromatography (HPLC), and nuclear magnetic resonance (NMR) (Figure 10a).^[^ ^29^, ^42^, ^61^, ^89^ ^]^ When selecting a suitable quantification method for urea, it is important to consider detection limits and potential interferences from the product matrix.^[^ ^90^ ^]^ In the following section, we review each detection method and discuss the potential pitfalls of each technique. For a reliable, reproducible, and accurate urea quantification, using a combination of at least two detection methods is highly recommended.

### 3.1 DAMO‐TSC Method

**Figure 9:**
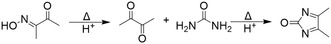
Proposed reaction of DAMO‐TSC method for urea detection.

The DAMO‐TSC colorimetric method is commonly used due to its shorter experimental time and lower cost compared to other methods.^[^ ^91^ ^]^ DAMO first decomposes into diacetyl under heat, which then reacts with urea in a highly acidic solution to form a yellow diazine product (**Figure** **9**).^[^ ^92^ ^]^

**Figure 10:**
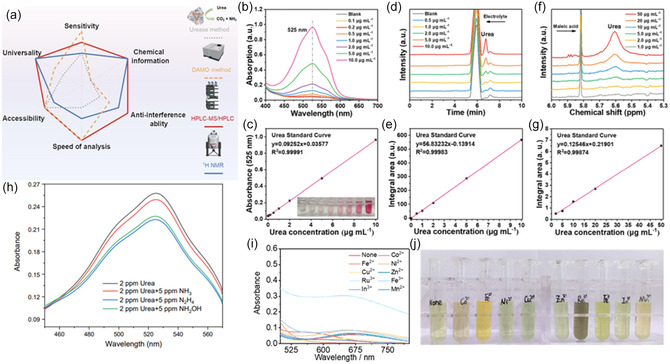
a) The comparison of four urea detection methods. Reproduced with permission.^[^ ^90^ ^]^ Copyright 2023, Elsevier. b) Proposed quantitative steps of photo‐/electrocatalytic urea synthesis based on the M‐DAMO‐TSC method. Measurement data and corresponding standard curve of urea determined by b,c) DAMO‐TSC; d,e) HPLC; and f,g) NMR methods. Reproduced with permission.^[^ ^92^ ^]^ Copyright 2023, Wiley‐VCH. h) UV‐vis absorption spectra of 2.0 ppm urea with 5.0 ppm N_2_H_4_, NH_3_, and NH_2_OH quantified by diacetyl monoxime method. i) UV‐vis spectra of indophenol blue method for ammonia obtained after the decomposition of 2 ppm urea with 25 ppm metal ions, and j) the corresponding photography of color substance. Reproduced with permission.^[^ ^90^ ^]^ Copyright 2023, Elsevier.

Phosphoric acid and thiosemicarbazide act as stabilizers, as the pink target product (4,5‐dimethyl‐2H‐imidazole‐2‐one), with absorption at 525 nm, is sensitive to light (**Figure** **10**b,c).^[^ ^90^, ^92^ ^]^ The presence of ferric ions enhances the absorbance of the colorimetric product.^[^ ^91^ ^]^ DAMO‐TSC method has been reported with a limit of detection of 0.26 ppm in pure water and 0.14 ppm in 0.2 M KHCO_3_. It is recommended that the DAMO‐TSC solution is remade every month, as the solution degrades over time.^[^ ^92^ ^]^ The DAMO‐TSC reaction typically occurs at 100 °C, as the key reagents are stable at this temperature. Temperatures above 100 °C can cause reagent degradation, reducing reactivity and leading to inconsistent color development.^[^ ^93^ ^]^ Lower temperatures (37, 45, and 60 °C) are impractical due to minimal color development.^[^ ^94^ ^]^ Once the DAMO‐TSC is performed, UV‐vis detection should be conducted promptly, as urea also decomposes over time.^[^ ^92^ ^]^ While DAMO‐TSC is accessible and provides a low limit of detection, further research is needed to address by‐product interference. For this method, the presence of NO_2_^−^ must be considered as it will affect the absorbance. Wang et al. discovered that when NO_2_^−^ concentration was less than 20 ppm in a 2.0 ppm urea solution, the results yielded an error range of ±7.5%. NO_2_^−^ concentrations over 20 ppm resulted in a significant fading due to the competing redox reaction between NO_2_^−^ and chromogenic product. The product of this reaction has an absorption of 488 nm, with the intensity increasing with the concentration of NO_2_^−^. To mitigate the interference of NO_2_^−^, Wang et al. demonstrated that adding sulfamic acid and hydrochloric acid to the solution effectively consumes NO_2_^−^ with a urea detection error of less than 5% for NO_2_^−^ concentrations up to 50 ppm.^[^ ^92^ ^]^ 5.0 ppm of other byproducts hydrazine (N_2_H_4_), NH_3_ and NH_2_OH, generated a lighter solution when compared to the pristine urea solution specifically (Figure 10h). This suggests that the concentration of byproducts, specifically NO_2_^−^, should be determined first, using methods such as ion chromatography or the Griess reagent.^[^ ^95^, ^96^ ^]^ Second, KHCO_3_, a commonly used buffer electrolyte for the C—N coupling reaction, requires careful pH adjustment of the DAMO‐TSC solution, as this colorimetric reaction occurs under highly acidic conditions. Third, because the reaction is light sensitive, it must be protected from sunlight and carried out in dark conditions and/or using amber vials.^[^ ^90^ ^]^

### 3.2 Urease Method

In the urease decomposition method, the concentration of NH_3_ in the solution needs to be first determined using the colorimetric indophenol blue method.^[^ ^97^, ^98^ ^]^ An additional solution is prepared by mixing urease with the analyte. Urease, an enzyme, then hydrolyzes urea into two NH_3_ and one CO_2_ molecule.^[^ ^99^ ^]^ The concentration of NH_3_ from the hydrolysis is then measured via the indophenol blue method. The final concentration of the urea is determined through the nitrogen atom balance (Equation (1))

(1) $$n_{\text{urea}}=2\times n_{{\text{NH}}_{3}}$$

The catalytic nature of urease decomposition depends on factors such as temperature and initial concentration of urea and urease.^[^ ^100^ ^]^ It was found that activity declines by 30% after 30 days at 4 °C; therefore, using a fresh solution is recommended.^[^ ^101^ ^]^ Additionally, the source of urease (e.g., jack beans, broad beans, bacteria) should be investigated, as it may slightly affect the optimal conditions. Optimal activity occurs between 30 and 60 °C, with denaturation at higher temperatures and reduced activity at lower temperatures.^[^ ^102^, ^103^ ^]^ At a given pH, any decrease in urea or urease concentration reduces ammonia production. Optimal activity occurs between pH 7 and 8, with denaturation in extreme acidic or alkaline conditions.^[^ ^101^, ^104^ ^]^ For practical purposes, the reaction can proceed for 30 min to 2 h, depending on the initial substrate concentration. While longer maturation times have minimal impact, shorter times may yield inaccurate results unless a higher enzyme concentration is used.^[^ ^105^ ^]^ While this detection may be more accurate than DAMO‐TSC due to minimal by‐product interference, it cannot distinguish between other C—N coupling species, such as methylamine or acetamide, resulting in an error bar within ±10%.^[^ ^90^ ^]^

#### 3.2.1 Indophenol Blue

**Figure 11:**
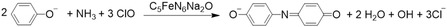
Proposed reaction mechanism of the Indophenol blue method for ammonia detection.

The indophenol blue method is a widely used detection method for NH_3_ quantification.^[^ ^98^ ^]^ The reaction proceeds via the Berthelot reaction, producing a blue‐colored indophenol product that absorbs light at 655 nm (**Figure** **11**).^[^ ^18^, ^106^ ^]^ First, NH_3_ reacts with a sodium hypochlorite solution, acting as an oxidizing agent to yield monochloroamine (NH_2_Cl). The pH of the solution must be above 9.25 to ensure the complete conversion of ammonium ions (*pK*_a_ = 9.25) to NH_3_.^[^ ^107^ ^]^ Benzoquinol chloramine is generated by the reaction between NH_2_Cl and the phenol group. However, the pH must be carefully controlled, as deprotonation of the phenol group (*pK*_a_ = 9.98) to a phenate ion in overly basic conditions inhibits the coupling reaction with NH_2_Cl. A second phenol group then couples with benzoquinol chloramine or its resonance form, benzoquinone chloramine, forming the indophenol blue complex. The dye remains stable up to pH 11.5, as NH_2_Cl begins to degrade beyond this pH.^[^ ^108^ ^]^ To ensure maximum absorbance, the solution should maintain a pH between 9.5 and 11. Temperature is critical for the Berthelot reaction kinetics, as temperatures below 10 °C result in lower absorbance of the colorimetric product. While 40 °C is often proposed as the optimal temperature for color development, conflicting reports suggest that 37 °C may be more optimal.^[^ ^109^, ^110^ ^]^ The reaction temperature also influences the maturation time (45 min to 2 h) required for optimal absorbance.^[^ ^111^ ^]^ Furthermore, when using this method, it is important to consider the potential leaching of metal ions from the electrode. At concentrations exceeding 25 ppm, Co^2+^, Fe^2+^, and Mn^2+^ can lead to an underestimated concentration of urea, as these metals inhibit urea hydrolysis by urease.^[^ ^96^, ^112^ ^]^ In contrast, Ru^3+^ may lead to an overestimation of urea concentration, as this ion reacts chemically with the colorimetric reagent (Figure 10i,j).^[^ ^96^, ^112^ ^]^

### 3.3 HPLC

HPLC is an analytical technique that identifies and quantifies compounds present in the analyte, with a typical detection limit greater than 0.5 ppm.^[^ ^92^ ^]^ This method is more accurate than the colorimetric methods discussed above, as it eliminates interferences from metal ions and side reaction products (Figure 10d,e).^[^ ^76^ ^]^ However, this technique is not as time efficient as colorimetric testing. Additionally, the speed and simplicity of colorimetric tests, particularly DAMO‐TSC, make them more appealing than HPLC as a primary detection method.

### 3.4 NMR

Like HPLC, NMR is an analytical technique used to identify and quantify urea in solution. For urea detection, deuterated dimethyl sulfoxide (DMSO‐d) is a suitable solvent, as other common ^1^H‐NMR solvents such as deuterium oxide (D_2_O), deuterated methanol (CD_3_OD), and deuterated acetone (C_3_D_6_O) undergo rapid hydrogen–deuterium exchange with the amine group of urea. While the chemical shift of DMSO‐d (2.5 ppm) does not overlap with that of urea (5.6 ppm), the water present in the sample does (4.6 ppm) (Figure 10f,g).^[^ ^90^ ^]^ Consequently, a water suppression mode must be used to prevent interference, which may weaken the urea peak intensity and lengthen the test duration. For accurate results, a minimum of 1000 scans on a 500 MHz NMR spectrometer is required, and the urea concentration should be greater than 5 ppm.^[^ ^92^ ^]^ NMR is currently considered the least attractive primary detection method compared to other techniques, due to its longer experiment duration and higher detection limit.

## 4 Electrochemical Designs

### 4.1 Electrochemical Cells

Two common types of electrochemical cells—H‐cells and flow cells—differ in design and application, resulting in variations in performance factors such as current density, selectivity, and catalyst stability.^[^ ^113^ ^]^

### 4.2 H‐Cell

H‐cells are a simple electrochemical setup, where gases (i.e., N_2_, CO_2_, etc.) are bubbled continuously into the electrolyte in the respective chamber. The cathodic and anodic chambers are separated with an ion exchange membrane to isolate the respective reaction taking place in the compartments, with this reaction occurring on the working electrode surface. The membrane allows for a selective ion flow between the chambers, while preventing the mixing of products. The reference electrode, such as Ag/AgCl or Hg/HgO, serves as a constant and known potential where the potential of the working electrode is measured. Counter electrodes, such as platinum foil or graphite rods, complete the electrical circuit, allowing current to flow.

### 4.3 Flow Cell

Flow cells consist of anode and cathode compartments, which may be separated by a membrane depending on if it's a single or two‐compartment flow cell. To minimize ohmic loss, the distance between the electrodes is usually less than 1 mm.^[^ ^114^ ^]^ The cathode is composed of a catalyst layer (CL), microporous layer (MPL), and gas diffusion layer (GDL). The CL is where the electrochemical reaction proceeds between the liquid and gas phases. The electrolyte flows to the CL through specially designed channels which ensures uniform distribution of the reactant(s) where the flow rate can be varied. The MPL facilitates a uniform transport of reactant gases to the CL from the GDL, while the hydrophobic properties regulate the flow of electrolyte to the CL. Additionally, the MPL gives structural support to the flooding of the GDL.^[^ ^115^ ^]^ A crucial parameter is to limit flooding of the cathodic chamber through hydrophobic properties to maintain the integrity of mass/charge transport.

Due to the mass transport and scale limitations of H‐cells, flow cells may offer a more optimal platform for C—N coupling. The gas reactants (such as CO_2_, N_2_, etc.) are directly involved at the electrode surface, which is advantageous compared to H‐cells, as these gases have poor solubility in water.^[^ ^116^, ^117^ ^]^

### 4.4 Polymeric Binders

In addition to the electrolyzer setup, other parameters—such as the polymer binder used for cathode fabrication—warrant future systematic studies to better understand their impact on the C—N coupling reaction. Binders like Nafion are commonly employed to attach catalysts to support materials^[^ ^118^, ^119^, ^120^ ^]^ Comparative studies of various polymers, such as polytetrafluoroethylene, polyvinyl alcohol, and polyvinylidene fluoride, could provide insight into the optimal binder material.^[^ ^120^ ^]^ Other critical considerations include ensuring that the binder is cost‐effective, chemically stable, and durable across a range of pH values.^[^ ^121^ ^]^

### 4.5 Cell Membranes

Membranes are crucial components of electrolyzers, serving as selective barriers that allow anodic and cathodic reactions to occur by controlling the transport of species in the solution. In electrochemical reactions, the choice of membrane depends on factors such as the type of ions being transported (i.e., anions or cations), pH, and desired efficiency. Commonly used membranes include proton exchange membranes (PEM) and anion exchange membranes (AEM). PEMs facilitate the transport of protons through the hydration of sulfonic acid groups, while blocking other species in the solution. Typical PEMs, manufactured with polymers like Nafion, must exhibit high proton conductivity, chemical stability, and mechanical strength. AEMs, on the other hand, enable the transport of anions such as hydroxide ions (OH^−^), while preventing cations and nonionic species from crossing the membrane. However, AEMs may not be ideal for C—N coupling, as the CO_2_ saturation in an H‐cell under alkaline conditions can lead to the formation of (bi)carbonates, which negatively impacts CO_2_ utilization and, consequently, performance. Challenges remain in improving the conductivity and ion selectivity of membranes, but further advancements in these areas could lead to the development of an optimal membrane.^[^ ^82^, ^83^ ^]^ Additionally, alternative membranes, such as bipolar membranes, could be explored for their impact on the C—N coupling reaction, as they show promise for industrial‐scale CO_2_ electrolysis.^[^ ^122^ ^]^

## 5 Summary and Outlook

Electrocatalytic synthesis of urea via the reaction between CO_2_ and nitrogen sources such as NO_3_^−^, NO_2_^−^, NO and offers a promising alternative to energy‐intensive urea production. This review aims to enhance understanding of C—N coupling progress by comparing various nitrogen sources and their advanced catalysts as well as quantification methods. Key strategies of catalyst design applicable across nitrogen feedstocks—such as tuning oxygen vacancies, doping, alloying, electronic surface modification, and constructing heterostructures—show potential for optimizing the process. However, as a relatively new research area that has only recently gained momentum, significant advancements are needed to elucidate mechanistic pathways and improve the performance factors such as FE, yield rate, and current density before transitioning C—N coupling from laboratory research to industrial applications. For urea to be produced on an industrial scale through the C—N coupling reaction, it has been suggested that the catalyst must achieve an FE of 73.24%, and a current density greater than 100 mA cm^−2^, with an electricity cost of $0.03 kW h^−1^.^[^ ^121^ ^]^ Expanding on the discussions above, the following prospects for future research aimed at developing practical and economically viable electrocatalytic systems for C—N coupling are highlighted. With the improvement of activity and selectively, C—N coupling offers a promising, sustainable commodity chemical production route.

### 5.1 Elucidating Mechanistic Pathways

While the mechanistic pathway varies with the nitrogen source and catalyst, understanding the reaction is critical for designing effective and selective catalysts for urea production. Although significant experimental and theoretical progress has been made, a conclusive chemical mechanism remains elusive. In situ characterization and spectroscopic methods, such as Raman, SR‐FTIR, and DEMS, have identified key intermediates; however, uncertainties around specific intermediates require further investigation through control experiments. Transient intermediates, often critical to the reaction mechanism, can sometimes be overlooked in in situ characterization, necessitating the need to combine experimental testing and in situ spectroscopy with computational studies and isotopic labeling. We would like to emphasize the importance of benchmarking the evaluation process of C—N coupling electrocatalysts by clearly including performance error bars. Additionally, using purified ^15^N‐labeled nitrogen sources for urea quantification, when necessary, is essential for accurately interpreting experimental data. In addition, distinguishing the potential‐determining step (PDS) from the rate‐determining step (RDS) is critical when investigating electrocatalytic pathways, especially in cases involving multiple steps where more than one PDS or RDS may exist. Improved mechanistic understanding will advance research into future catalyst design, enhancing their abilities to activate CO_2_ and nitrogen species efficiently, while also minimizing undesirable processes such as intermediate desorption.^[^ ^123^ ^]^

### 5.2 Optimizing Electrocatalytic Conditions

While the C—N coupling reaction occurs at the cathode, the anodic reaction must also be considered as well. The typical reaction at the anode is the oxygen revolution reaction (OER), a four‐proton, four‐electron process that requires a high overpotential due to sluggish reaction kinetics.^[^ ^124^ ^]^ Optimizing the anodic reaction to a less energy‐intensive process, such as methanol or hydrazine oxidation, could improve the economic viability of the process.^[^ ^125^ ^]^ Under cathodic conditions, modifying the local environment of the catalyst surface between liquid and gaseous species has proven effective for activating CO_2_ and nitrogen species.^[^ ^15^, ^126^, ^127^, ^128^ ^]^ To mitigate the HER, which lowers the FE and yield of urea, further evaluation of the pH conditions for C—N coupling in aqueous conditions is needed. As the neutral aqueous solution undergoes CO_2_ saturation, carbonic acid (H_2_CO_3_) is generated, releasing protons into the solution. H_2_CO_3_ rapidly dissociates, producing hydrogen and bicarbonate ions, acidifying the bulk solution (pH = 5.8). However, CO_2_ saturation into an aqueous electrolyte with the addition of 0.1 M KHCO_3_ (pH = 8.52) suppresses the generation of H_2_CO_3_ thus minimizing the solution acidification (pH = 6.81).^[^ ^129^ ^]^ Additionally, future studies on the influence of pH on the CO_2_RR toward the hypothesized critical carbon intermediate could aid in optimizing C—N coupling processes.^[^ ^126^, ^130^ ^]^

Performing the NO_3_RR in acidic environments may offer advantages over alkaline or basic electrolytes, as protons are readily available to hydrogenate NO_3_^−^.^[^ ^23^ ^]^ This could result in a higher conversion rate to NH_3_ while reducing undesirable NO_2_^−^ production, a common byproduct in C—N coupling. With several C—N coupling studies suggesting that the nitrogen intermediate is an NH_3_‐like precursor, a higher concentration of this species may promote an increase in urea production. However, a broader range of catalysts is needed, as many late‐transition metals (such as Cu, Fe, and Ni) are unstable in acidic conditions, and the environment is also favorable for H_2_ generation.^[^ ^15^, ^23^ ^]^ Similar to the CO_2_RR, the effects of buffers in the electrolyte can be compared as the pH of the solution increases over time, due to the proton consumption during ammonia production in the reduction reactions.^[^ ^131^ ^]^ While KCHO_3_ is a commonly used buffer for both CO_2_RR and C—N coupling, there is limited reporting on other buffers, such as phosphate‐buffered saline for C—N coupling.^[^ ^132^ ^]^

### 5.3 Exploring Alternative C—N Coupling Products

Expanding the focus of C—N coupling beyond the discussed nitrogen sources and CO_2_, the scope of commodity chemicals can extend pass urea to species such as formamide, acetamide, and amino acids (**Table** **5**). As knowledge of urea production pathways and alternative C—N products expands, it may provide insights into how these factors influence the desired C—N product, thereby enriching the catalytic catalog and advancing future studies.^[^ ^133^ ^]^ Additionally, as advancements are made in scaling up CO_2_RR for industrial use, other carbon products generated in these processes, such as CO and formic acid (HCOOH), could be repurposed for C—N coupling reactions.^[^ ^134^, ^135^ ^]^

**Table 5:** Reported electrocatalysts for alternative C—N products with various carbon and nitrogen sources.

Catalyst	Electrolyte	Electrochemical cell	*J*_product_ [mA cm^−2^]	Potential [V_RHE_]	FE [%]	Product	References
Cu_2_O NC	0.5 M NaOH, 0.2 M HCOOH, 0.02 NaNO_2_	H‐cell	35.1 mmol h^−1^ g_cat_^−1^	−0.4	29.7	Formamide	[156]
Ru_1_Cu SAA	1.0 M KOH + 1 M KNO_2_	H‐cell	5.5	−0.5	45.65	Formamide	[157]
Cu_2_O NC	0.5 M NaOH, 0.2 M CH_3_COOH, 0.02 NaNO_2_	H‐cell	–	−0.4	16.4	Acetamide	[156]
Cu NPs	1.0 M KOH	Flow cell	114	−0.68	38	Acetamide	[158]
Cu NPs	1.0 M KOH	Flow cell	2.2	−0.58	10	Acetamide	[159]
Cu–Hg	15 wt% H_2_SO_4_ aqueous solution, 0.25 M glyoxylic acid, 0.25 M NaNO_3_	H‐cell	–	−1.4 *V*_Ag/AgCl_	43.1	Glycine	[160]
Fe–N–C‐700	0.5 M Oxalic acid + 0.5 M NaNO_3_	H‐cell	106.4	−1.0	64.2	Glycine	[161]

### 5.4 Prospective Point Sources

Power plants may serve as attractive point sources for future industrial‐scale C—N coupling reactions for urea synthesis, as they emit both CO_2_ and NO_*x*_. For example, the New Madrid power plant in Marston, Missouri, emitted 15,489 tons of NO_*x*_ and 7,459,852 tons of CO_2_ in 2022.^[^ ^136^ ^]^ Ammunition plants may also be desirable point sources; for instance, in 2020, the Radford Army Ammunition Plant in Radford, Virginia, emitted 149,392 metric tons of CO_2_ and 84 metric tons of N_2_O.^[^ ^136^ ^]^ Additionally, wastewater released from this plant contained 5,957 tons of NO_3_^−^, providing multiple potential nitrogen species for synthesis from a single point source.^[^ ^137^ ^]^

## Conflict of Interest

The authors declare no conflict of interest.
